# Supplementary material for: Bacterial Endophyte Community Dynamics in Apple (Malus domestica Borkh.) Germplasm and Their Evaluation for Scab Management Strategies
Source: J Fungi (Basel). 2021 Oct 31;7(11):923. doi: 10.3390/jof7110923 (PMC8623955; doi:10.3390/jof7110923)
Supplement: Supplementary file 1 [file jof-07-00923-s001.zip › jof-1353186-supplementary.pdf]

Table S1: Details of the surveyed apple germplasm and isolation thereof

**L**	**S**	**P**	**P_l**	**I**	**S_v**	**F**
**A_g**	R_d	T	4.70	DS1, DS2, DS7	DS2	NWHA-1
		L	5.90	DS5, DS6		
		R	6.12	DS3, DS4, DS9, DS8	DS4	NWHA-2
	G_d	T	5.67	DS10, DS13, DS15, DS11, DS12	DS10, DS15, DS11	NWHA-3, WHA-4, NWHA-5
		L	5.97	DS14, DS16, DS19	DS14, DS16, DS19	NWHA-6 NWHA-7 NWHA-8
		R	7.27	DS17, DS18, DS20, DS23, DS24	DS17, DS18, DS23	NWHA-9, NWHA-10 NWHA-11
	R_c	T	6.31	DS21,DS22, DS25	DS25	NWHA-12
		L	6.02	DS26, DS29		
		R	6.89	DS28, DS30,DS31	DS31	NWHA-13
	G_r	T	6.33	DS27,DS32,DS35	DS35	NWHA-14
		L	5.49	DS34,DS37,DS38	DS33	NWHA-15
		R	6.55	DS33, DS36,DS39		
	F_z	T	5.07	DS41,DS42, DS45	DS45	NWHA-16
		L	6.19	DS40		
		R	7.12	DS43, DS44,DS46		
	M_j	T	6.32	DS49,DS51	DS51	NWHA-17
		L	7.83	DS48, DS53	DS48, DS53	NWHA-18, NWHA-19
		R	8.27	DS47,DS50	DS50	NWHA-20
**P_l**	R_v	T	5.36	DS52, DS57	DS57, DS54	NWHA-21, NWHA-22
		L	5.72	DS56,DS55		
		R	7.42	DS54		
	G_m	T	5.38	DS59,DS61	DS60	NWHA-23
		L	5.89	DS57,DS60		
		R	6.39	DS58,		
	M_b	T	6.48	DS63,DS71, DS75	DS63, DS71, DS75	NWHA-24,NWHA-25, NWHA-26
		L	7.29	DS67,DS72, DS74	DS74	NWHA-27
		R	7.88	DS64,DS66,DS73	DS66, DS73	NWHA-28,NWHA-29
	G_d	T	5.39	DS69, DS76	DS76	NWHA-30
		L	5.35	DS65, DS68		
		R	6.44	DS62, DS70,DS77	DS77	NWHA-31
	S_r	T	4.78	DS79, DS83	DS81	NWHA-32
		L	5.41	DS82, DS81		
		R	5.84	DS80, DS84,DS78	DS78	NWHA-33
	R_f	T	5.11	DS84, DS85, DS90	DS90	NWHA-34
		L	5.65	DS87, DS88	DS88	NWHA-35
		R	7.06	DS86, DS89, DS91		
**B_l**	P_s	T	6.48	DS93, DS97	DS93	NWHA-36
		L	6.09	DS95,DS98, DS99	DS95, DS99	NWHA-37, NWHA-38
		R	7.51	DS92, DS94, DS96, DS100	DS92, DS94, DS100	NWHA-39, NWHA-40, NWHA-41
	O_s	T	5.19	DS102, DS104, DS106	DS106, DS101	NWHA-42, NWHA-43
		L	5.92	DS105, DS107		
		R	6.38	DS101, DS103		
	G_v	T	5.29	DS108, DS114	DS113, DS111, DS115	NWHA-44, NWHA-45, NWHA-46
		L	6.71	DS112, DS113,		
		R	6.68	DS109, DS110, DS111, DS115		
	L_c	T	6.05	DS116, DS121, DS124	DS116, DS124	NWHA-47, NWHA-48
		L	6.39	DS119, DS120	DS119, DS120	NWHA-49, NWHA-50
		R	7.28	DS117, DS123, DS118,DS122	DS117, DS122	NWHA-51, NWHA-52
	M_n	T	6.11	DS127, DS128, DS133, DS134	DS128, DS133, DS134	NWHA-53, NWHA-54, NWHA-55
		L	5.89	DS130, DS131	DS130	NWHA-56
		R	7.69	DS125, DS126, DS129, DS132	DS125, DS129, DS132	NWHA-57, NWHA-58, NWHA-59
	F_n	T	7.21	DS135, DS143, DS144	DS135, DS144	NWHA-60,NWHA-61
		L	6.11	DS137, DS139, DS140	DS137, DS140	NWHA-62,NWHA-63
		R	7.96	DS136, DS138, DS141, DS142, DS145	DS136, DS141, DS142, DS145	NWHA-64, NWHA-65 NWHA-66, NWHA-67
**S_p**	A_b	T	5.67	DS146, DS148, DS152	DS152	NWHA-68
		L	6.49	DS149, DS150, DS154, DS155	DS149, DS155	NWHA-69, NWHA-70
		R	6.87	DS147, DS151, DS153	DS147	NWHA-71

*****L:Location;S: Sampling variety/cultivar; P: Plant part;P_l :Population $Log_{10} \times 10^5$ CFU/mL; I: Isolated strains; S_v:Strains with antifungal behavior; F: Final code of isolate; A_g: Anantnag; P_l: Pulwama; B_l: Baramula; S_n:Shopian; R_d:Red delicious; G_d: Golden delicious; R_c:Red chief; G_r: Gala Redlum; F_z: Fuji Zehn Aztech; M_j: Maharaji; R_v: Red velox; G_m: Gala mast; M_b: Malus floribunda; S_r: Summer red; R_f: Red fuji; P_s: Priscella; O_s: Oregon spur; G_v:Gavin; L_c: Lal cider; M_n:Manchurian; F_n:Fenna; A_b:Ambri; T:Twigs;L:Leaf; R:Root***

Table S2. Details of the sequenced microflora with antifungal behavior against *Venturia inaequalis*

Isolate	Bacterial species	Accession number	Max. Identity
NWHA-1	*Bacillus aryabhattai* strain DST scab	MH718825	1533/1533, (100%)
NWHA-2	*Pseudomonas veronii* strain DST scab2	MH718842	1520/1521, (99%)
NWHA-3	*Bacillus cereus* strain DST scab3	MH725572	1563/1563, (100%)
NWHA-4	*Bacillus amyloliquefaciens* strain DST scab4	MH725637	1553/1553, (100%)
NWHA-5	*Bacillus siamensis* strain DST scab5	MH732922	1533/1533, (100%)
NWHA-6	*Bacillus amyloliquefaciens* strain DST scab6	MH732929	1557/1557, (100%)
NWHA-7	*Bacillus velezensis* strain DST scab7	MH734811	1560/1560, (100%)
NWHA-8	*Bacillus siamensis* strain DST scab8	MH734812	1524/1524, (100%)
NWHA-9	*Bacillus aryabhattai* strain DST Scab9	MT150705	1533/1533, (100%)
NWHA-10	Bacillus subtilis strain DST10	MT150715	1543/1543, (100%)
NWHA-11	*Ochrobactrum pecoris* strain DST11	MT750122	1399/1399, (100%)
NWHA-12	*Paenibacillus amylolyticus* strain DST12	MT154258	1511/1511, (100%)
NWHA-13	*Paenibacillus tundrae* strain DST13	MT158221	1526/1526, (100%)
NWHA-14	*Paenibacillus xylanexedens* strain DST14	MT154260	1526/1526, (100%)
NWHA-15	*Rahnella aquatilis* strain DST15	MT154261	1505/1505, (100%)
NWHA-16	*Pseudomonas mosselii* strain DST16	MT154264	1430/1430, (100%)
NWHA-17	*Sphingomonas wittichii* strain DST17	MT165895	1498/1498, (100%)
NWHA-18	*Variovorax boronicumulans* strain DST18	MT163368	1483/1483, (100%)
NWHA-19	*Rhizorhabdus dicambivorans* strain DST19	MT163374	1470/1470, (100%)
NWHA-20	*Ramlibacter ginsenosidimutans* strain DST20	MT163375	1470/1470, (100%)
NWHA-21	*Pseudomonas veronii* strain DST21	MT166342	1503/1503, (100%)
NWHA-22	*Pseudomonas fluorescens* strain DST22	MT163392	1512/1515, (99.99%)
NWHA-23	*Pantoea agglomerans* strain DST23	MT176480	1503/1503, (100%0
NWHA-24	*Povalibacter uvarum* strain DST24	MT163394	1507/1508, (99.99%)
NWHA-25	*Enterobacter hormaechei* subsp. *xiangfangensis* strain DST25	MT163395	1500/1500, (100%)
NWHA-26	*Bacillus foraminis* strain DST26	MT176179	1533/1534, (99.99%)
NWHA-27	*Pseudomonas chlororaphis* strain DST27	MT163397	1365/1365, (100%)
NWHA-28	*Pseudomonas psychrophila* strain DST28	MT163428	1537/1537, (100%)
NWHA-29	*Pseudomonas fragi* strain DST29	MT176180	1458/1462, (99.99%)
NWHA-30	*Bacillus flexus* strain DST30	MT163343	1500/1500, (100%)
NWHA-31	*Paenibacillus lautus* strain DST31	MT163448	1499/1499, (100%)
NWHA-32	*Paenibacillus peoriae* strain DST32	MT163453	1515/1515, (100%)
NWHA-33	*Bacillus subterraneus* strain DST33	MT163452	1522/1522, (100%)
NWHA-34	*Paenibacillus polymyxa* strain DST34	MT163461	1519/1519, (100%)
NWHA-35	*Enterobacter cloacae* strain DST35	MT163464	1481/1481, (100%)
NWHA-36	*Klebsiella pneumoniae* strain DST36	MT163466	1498/1498, (100%)
NWHA-37	*Cedecea neteri* strain DST37	MT176181	1463/1465, (99.99%)
NWHA-38	*Enterobacter cloacae* strain DST338	MT176765	1516/1516, (100%)
NWHA-39	*Leclercia adecarboxylata* strain DST39	MT176482	1449/1449, (100%)
NWHA-40	*Enterobacter ludwigii* strain DST40	MT176481	1454/1454, (100%)
NWHA-41	*Bacillus amyloliquefaciens* strain DST41	MT176484	1473/1473, (100%)
NWHA-42	*Bacillus velezensis* strain DST42	MT176485	1380/1380, (100%)
NWHA-43	*Pseudomonas synxantha* strain DST43	MT176487	1245/1249, (99.99%)
NWHA-44	*Pseudomonas libanensis* strain DST44	MT176512	1518/1518, (100%)
NWHA-45	*Pseudomonas libanensis* strain DST45	MT176513	1516/1516, (100%)
NWHA-46	*Bacillus amyloliquefaciens* strain DST46	MT176523	1427/1427, (100%)
NWHA-47	*Pseudomonas grimontii* strain DST47	MT176524	1470/1472, (99.99%)
NWHA-48	*Bacillus siamensis* Strain DST48	MT176526	1467/1467, (100%)
NWHA-49	*Bacillus amyloliquefaciens* strain DST49	MT176527	1509/1509, (100%)
NWHA-50	*Bacillus subtilis* strain DST50	MT176530	1499/1499, (100%)
NWHA-51	*Curvibacter gracilis* strain DST51	MT254903	1484/1484, (100%)
NWHA-52	*Delftia acidovorans* strain DST52	MT254906	1515/1515, (100%)
NWHA-53	*Enterobacter asburiae* strain DST53	MT180564	1406/1408, (99.99%)
NWHA-54	*Enterobacter ludwigii* strain DST54	MT180566	1492/1492, (100%)
NWHA-55	*Salinivibrio costicola* strain DST55	MT180567	1483/1487, (99.99%)
NWHA-56	*Vibrionaceae bacterium* strain DST56	MT180709	1466/1466, (100%)
NWHA-57	*Leclercia adecarboxylata* strain DST57	MT180610	1514/1514, (100%)
NWHA-58	*Enterobacter asburiae* strain DST58	MT180710	1424/1424, (100%)
NWHA-59	*Ochrobactrum pecoris* strain DST59	MT180711	1389/1389, (100%)

NWHA-60	*Ochrobactrum tritici* strain DST60	MT180712	1474/1474, (100%)
NWHA-61	*Ochrobactrum anthropi* strain DST61	M7180723	1445/1445, (100%)
NWHA-62	*Rahnella aquatilis* strain DST62	MT180745	1472/1474, (100%)
NWHA-63	*Curtobacterium herbarum* strain DST63	MT186202	1492/1492, (100%)
NWHA-64	*Pseudomonas libanensis* strain DST64	MT271984	1518/1518, (100%)
NWHA-65	*Bacillus simplex* strain DST65	MT186229	1525/1525, (100%)
NWHA-66	*Glutamicibacter endophyticus* strain DST66	MT186238	1583/1583, (100%)
NWHA-67	*Glutamicibacter halophytocola* strain ST67	MT186239	1524/1524, (100%)
NWHA-68	*Comamonas terrigena* strain DST68	MT186246	1466/1466, (100%)
NWHA-69	*Delftia acidovorans* strain DST69	MT186247	1509/1509, (100%)
NWHA-70	*Micrococcus yunnanensis* strain DST70	MT186248	1493/1493, (100%)
NWHA-71	*Pseudomonas fluorescens* strain DST71	MT186249	1470/1470, (100%)
